# Influence of Liposomes’ and Lipoplexes’ Physicochemical Characteristics on Their Uptake Rate and Mechanisms by the Placenta

**DOI:** 10.3390/ijms23116299

**Published:** 2022-06-04

**Authors:** Louise Fliedel, Khair Alhareth, Johanne Seguin, Marwa El-Khashab, Audrey Chissey, Nathalie Mignet, Thierry Fournier, Karine Andrieux

**Affiliations:** 1 Université Paris Cité, CNRS, INSERM, Unité des Technologies Chimiques et Biologiques pour la Santé (UTCBS), F-75006 Paris, France; louise.fliedel@etu.u-paris.fr (L.F.); khairallah.alhareth@u-paris.fr (K.A.); johanne.seguin@u-paris.fr (J.S.); marwaelkhashab94@gmail.com (M.E.-K.); nathalie.mignet@u-paris.fr (N.M.); 2 Université Paris Cité, INSERM, Pathophysiology and Pharmacotoxicology of the Human Placenta, Pre and Postnatal Microbiota unit (3PHM), F-75006 Paris, France; audrey.chissey@u-paris.fr (A.C.); thierry.fournier@inserm.fr (T.F.)

**Keywords:** liposomes, lipoplexes, ex vivo model, cellular uptake, endocytosis, placental explants

## Abstract

Pregnant women are still considered as drug orphans. Developing new medications for pregnancy complications is an urgent need. Nanomedicines seem to be a promising approach to control the biodistribution of drugs to ensure both the mother’s and the fetus’ safety. Understanding the interaction between nanoparticles and the placental barrier is a key factor to the success of the development of nanomedicines for pregnant women. In this study, we evaluated the behavior of fluorescent PEGylated liposomes and lipoplexes in human placental tissue using in vitro and ex vivo models, BeWo cell culture and suspended villous placental explants, respectively. Fluorescent based analytical tools such as Fluorescence activated cells sorting (FACS), confocal microscopy and HPLC coupled to fluorescence detection were used to assess liposomes penetration and their endocytosis mechanisms in the placenta. First, no influence of the PEGylation density was observed on the cellular internalization of liposomal formulations using both models. The comparison between neutral and cationic liposomes exhibits a significant higher internalization of the cationic formulation compared to the neutral ones. In addition, the HPLC quantification of the fluorescent liposomes in human villous explants demonstrated an increase of cationic liposomes uptake with increasing incubation concentrations. Similar uptake of cationic liposomes and lipoplexes, containing the same cationic lipid, the DMAPAP but with an overall neutral surface charge, was observed and evidenced the higher effect of composition than charge surface on trophoblast penetration. Moreover, both cationic liposomes and lipoplexes exhibited an endocytosis mechanism of internalization via pathways implicating dynamin. These data highlight the key role of the liposome’s lipid composition and the possibility to modulate their internalization in the placenta by adjusting their design.

## 1. Introduction

Women can experience various diseases during pregnancy and need treatment accordingly. Finding new therapeutics for acute and chronic diseases represents a challenge and especially for pregnant women therapy. Most approved drugs after clinical trials fail to have consistent and appropriate data regarding the use of medication during pregnancy [1,2]. Therefore, providing safe and effective treatments for pregnant women with guarantee of their offspring’s safety could be achieved by controlling the drug distribution and preventing its transplacental passage. Nanomedicine can be a potential therapeutic approach to use during pregnancy [3]. Controlled drug delivery systems, such as liposomes, tend to overcome conventional dosage forms limitations by improving therapeutic efficacy and decreasing associated toxicity. The rising interest for nanoparticle-based medicine development for pregnant women asks the question of their biological evaluation [4].

The key feature during pregnancy lies within a specific organ, the placenta. It is a transitory organ, connecting the maternal and the fetal blood circulations. It provides nutrients and oxygen to the fetus and allows the elimination of fetal waste. But Active Pharmaceutical Ingredients (API) administered to the mother can cross the placenta and reach the fetal side leading to severe outcomes.

Pivotal studies, focusing on developing new treatments for pregnancy-associated disorders, rely on nanocarriers use for API delivery [4]. Therefore, it is important to better understand parameters, which influence the placental uptake of nanoparticles, in order to predict their potential as a carrier of API to limit its transplacental passage [5,6]. To go deeper in the understanding of nanoparticles placental uptake, endocytosis mechanisms have to be studied, as nanoparticles penetrate cells through endocytosis pathways [7]. These pathways are well-described among various cell types, but data are missing for placental cells. Findings on those internalization mechanisms could help empower the use of nanoparticles for a delicate clinical situation such as pregnancy. This is a preliminary work to study how model nanocarriers can interact with the placental barrier by means of relevant in vitro and ex vivo placental models before transferring to actual therapy. The nanocarriers’ formulations should be optimized regarding the encapsulated API and therefore the impact of drug loading in liposomes should be studied case by case in the delicate context of pregnancy.

In this study, factors which are known to influence the cellular uptake of liposomes (content of PEG, surface charge, lipid composition) were evaluated on an in vitro model of human trophoblast cell line (BeWo cells) and an ex vivo model of suspended human villous placental explants. Fluorescently labeled neutral liposomes, cationic liposomes and lipoplexes (combination of cationic liposomes and a model siRNA) were manufactured and their placental uptake was investigated. Protocols for qualitative and quantitative analysis of internalization were optimized using appropriate analytical tools, such as confocal microscopy, HPLC-fluorescence detection and fluorescence activated cells sorting (FACS). Endocytosis inhibitors were used to determine the mechanisms of liposomal internalization by BeWo cells.

## 2. Results and Discussion

### 2.1. Liposomes and Lipoplexes Characterization

As shown in Table 1, the cationic liposomes had a size < 110 nm and neutral liposomes had size < 130 nm, both with narrow size distribution (PdI under 0.2). The fluorescent cationic liposomes had a positive charge > 30 mV, while neutral liposomes showed a charge between 0 and −10 mV.

Lipoplexes containing 5% of DSPE-PEG_2000_ exhibited a hydrodynamic diameter around 200 nm and a PDI under 0.2, which was expected as it has already been demonstrated in previous studies [8,9]. Formulations containing only 1% DSPE-PEG_2000_ showed higher PdI and the formulation was not stable during storage. Concerning the zeta potential, formulations exhibited a surface charge around 7–11 mV, which was considered as neutral. Even if the lipoplexes were composed of 50% of cationic lipids, the overall surface charge stayed neutral, thanks to the presence of siRNA and the addition of sodium alginate in the formulation which neutralizes the cationic charges.

No influence of the percentage of DSPE-PEG_2000_ (1 or 5%) has been observed on the hydrodynamic diameter and zeta potential of formulations as expected [10].

Then, the behavior of the formulations was assessed by ex vivo and in vitro placental models.

### 2.2. Cytotoxicity Assay of Liposomes and Lipoplexes

The cytotoxicity assay was performed on a controlled number of BeWo cells per well. The control was cells incubated with dilutions of NaCl 150 mM to ensure the liposome-dispersing solvent has no toxic effect on cells.

**Figure 1:**
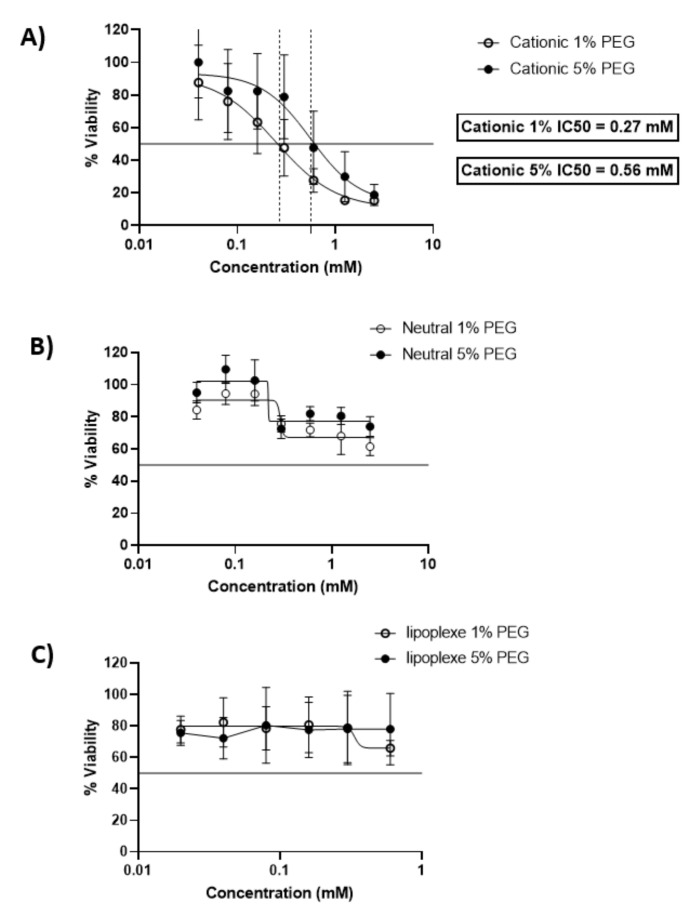
Dose-response curves expressing the viability of BeWo cells after incubation with cationic liposomes (**A**), neutral liposomes (**B**) and lipoplexes (**C**) for 24 h. The x-scale of the lipoplexes MTT (**C**) is different from the liposome graphs (**A**,**B**). The data represent the results of MTT assay and are expressed as average % cell viability ± SEM against the concentration of the liposomes in mM. Error bars indicate standard deviation for n = 3 formulations.

After 24 h of incubation cationic liposomes reduced cellular viability with an IC50 of 0.27 mM and 0.56 mM for 1 and 5 % PEG respectively. 80% of cells were viable under concentrations of 0.03 mM for both formulations. The neutral liposomes showed a better viability of the cells since the formulations with 5 % of PEG preserved 80 % viability of the cells above 0.15 mM, while the viability of the cells decreased below 80% with formulations containing 1% of PEG above lipid concentrations of 0.25 mM. Lipoplexes exhibited a low toxicity on cells and allowed 80% viability of cells for all concentrations tested (Figure 1).

These results are in accordance with previous studies. Neutral liposomes exhibit a moderate toxicity over 24 h of incubation with BeWo cells, while cell viability decreases rapidly in the presence of cationic liposomes [11]. Concerning lipoplexes the cytotoxicity was reduced compared to the cationic liposomes and had a similar profile as the neutral liposomes, exhibiting the non-toxic profile of lipid nanoparticle with a neutral surface charge [12].

### 2.3. Assessment of Charge Effect on Liposomes’ Internalization by Placental Cells

#### 2.3.1. Qualitative Evaluation on Human Villous Placental Explants

To assess the influence of the surface charge on the interaction of neutral and cationic liposomes with placenta, formulations were incubated with placental explants. For this experiment, only liposomes containing 5% of PEG were incubated for 24 h at increasing concentrations of 0.15 mM, 0.3 mM, and 0.5 mM. The range of concentrations was determined as non-toxic on an ex vivo model of placental explants in a previous work [13].

**Figure 2:**
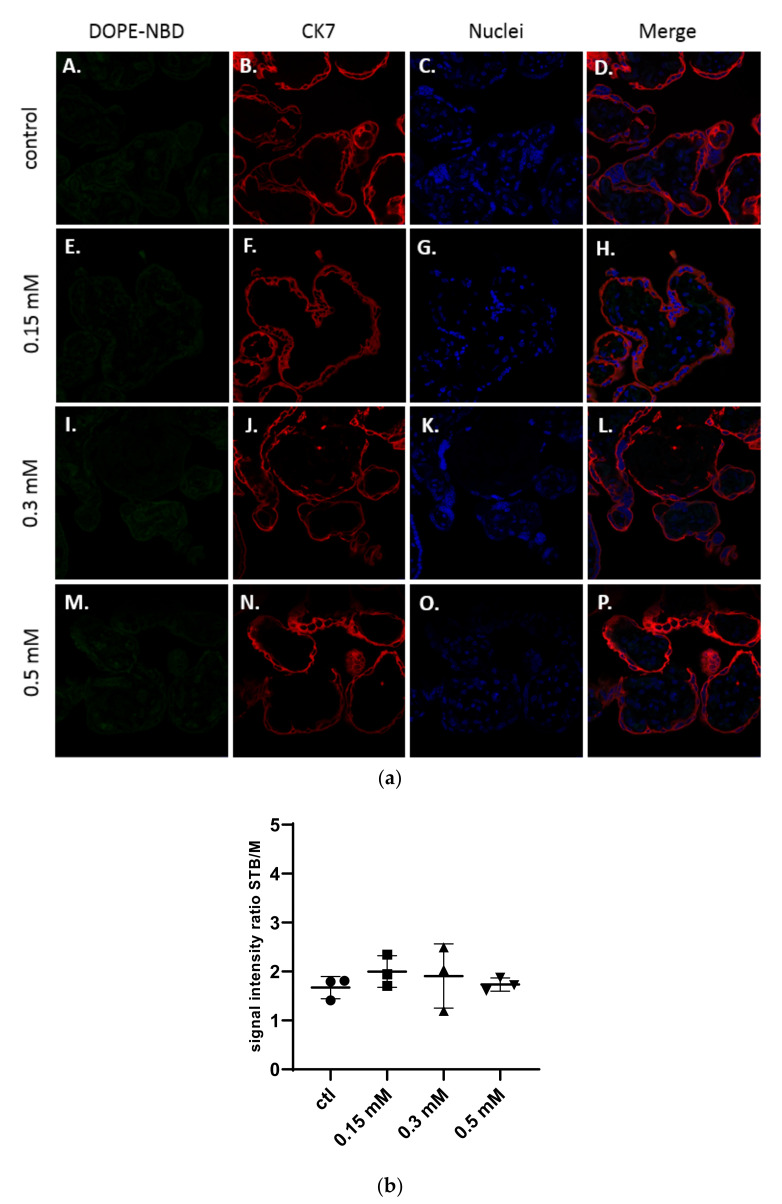
(**a**) Confocal images of term placental villi incubated with medium (A–D), and neutral liposomes (5 % DSPE-PEG_2000_) at various concentrations: 0.15 mM (E–H); 0.3 mM (I–L); 0.5 mM (M–P). Green: liposomes (DOPE-NBD); Red: syncytiotrophoblast (CK7); Blue: nuclei (DAPI); (**b**) Semi quantitative analysis of green fluorescence intensity ratio between the syncytiotrophoblast region and mesenchymal axis of placental villi analyzed with confocal microscopy and treated with neutral liposomes at different concentrations (n = 3 villi).

As shown in Figure 2a, structures of the placenta were observed thanks to the specific staining of the syncytiotrophoblast membranes by a primary antibody targeting the cytokeratin-7 (CK7), a specific marker of these structures, labelled with a secondary antibody Alexa Fluor^®^ 555 in red and of the DNA in blue with a DAPI staining. The liposomes were detected in green thanks to the presence of the fluorescent lipid DOPE-NBD at 1% inside the formulations.

No presence of neutral liposomes was detected within the explant structures after confocal microscopy analysis (Figure 2a). The semi quantitative analysis of the fluorescence intensity of images in comparison with the control confirmed those results (Figure 2b).

**Figure 3:**
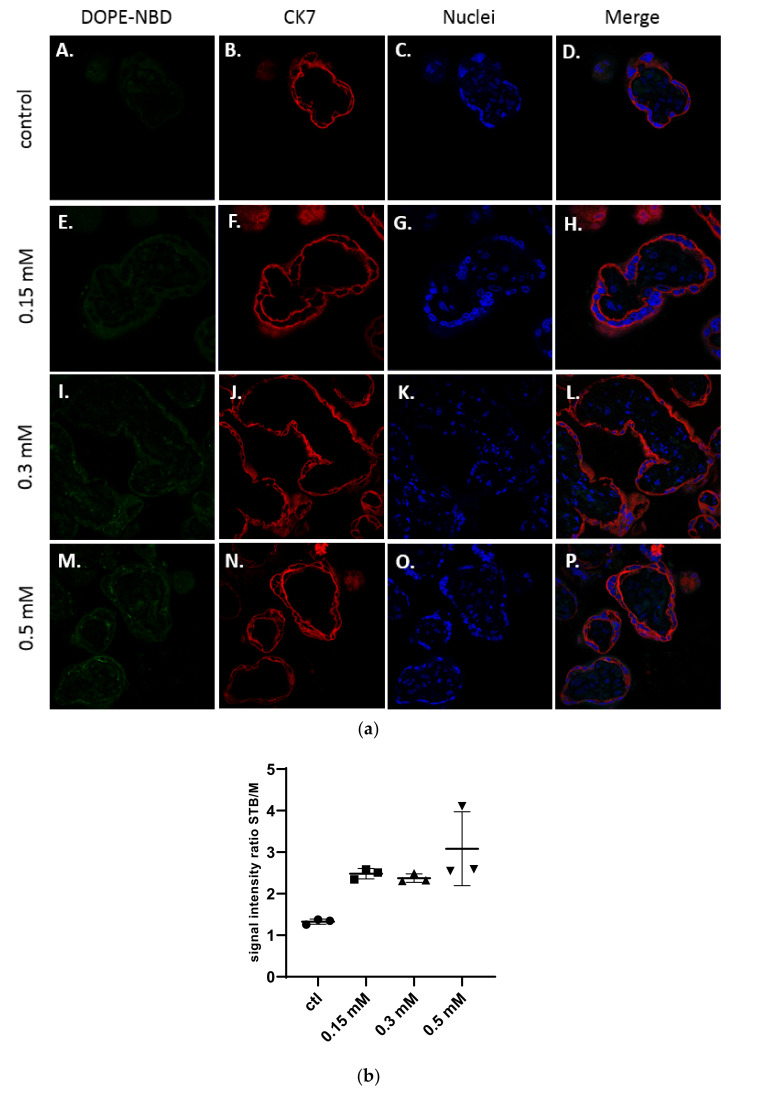
(**a**) Confocal microscopy images of placental chorionic villi (x63) representing explants incubated with medium (A–D), and cationic liposomes (5 % DSPE-PEG_2000_) at various concentrations: 0.15 mM (E–H); 0.3 mM (I–L); 0.5 mM (M–P). Green: liposomes (DOPE-NBD); Red: syncytiotrophoblast (CK7); Blue: nuclei (DAPI); (**b**) Semi quantitative analysis of spinning disk microscopy of the green fluorescence intensity ratio between the syncytiotrophoblast region and mesenchymal axis of placental villi treated with cationic liposomes at different concentrations (n = 3 villi).

In Figure 3a, confocal microscopy images exhibited the presence of cationic liposomes within the layer of the syncytiotrophoblast, but no fluorescence has been observed within the mesenchymal axis, suggesting these liposomes were not crossing to the fetal side. The semi quantitative analysis in Figure 3b confirmed the results. Moreover, no dose-effect on the internalization of cationic liposomes into explants could be evidenced.

These qualitative results were then compared to two different qualitative analyses: HPLC quantification of the DOPE-NBD in villous placental explants and Fluorescence Activated Cell Sorting (FACS) analysis on BeWo cells.

#### 2.3.2. Quantitative Assessment of Liposomes’ Uptake by Placental Cells Using HPLC and FACS

##### HPLC Dosage of Fluorescent Liposomes Inside Human Villous Placental Explants 

Formulations were incubated for 24 h with placental villous explants, then after collection explants were frozen at −80°C and liquid-liquid extraction was performed to retrieve lipids present within the samples. HPLC quantification of DOPE-NBD was performed on samples using the previously described method [14].

Quantification of DOPE-NBD has been done in each villus (Table 2). Since each villus had specific shape and weight within a same placenta, the quantification of DOPE-NBD inside samples was normalized using the weight of each villus. Results showed no significant uptake for neutral liposomes, whereas the percentage of uptake increases with the concentration of incubation for cationic liposomes, evidencing a dose-effect response. These HPLC data confirmed and completed the previous results obtained by confocal microscopy.

##### FACS Evaluation of Liposomes’ Internalization in BeWo Cells

**Figure 4:**
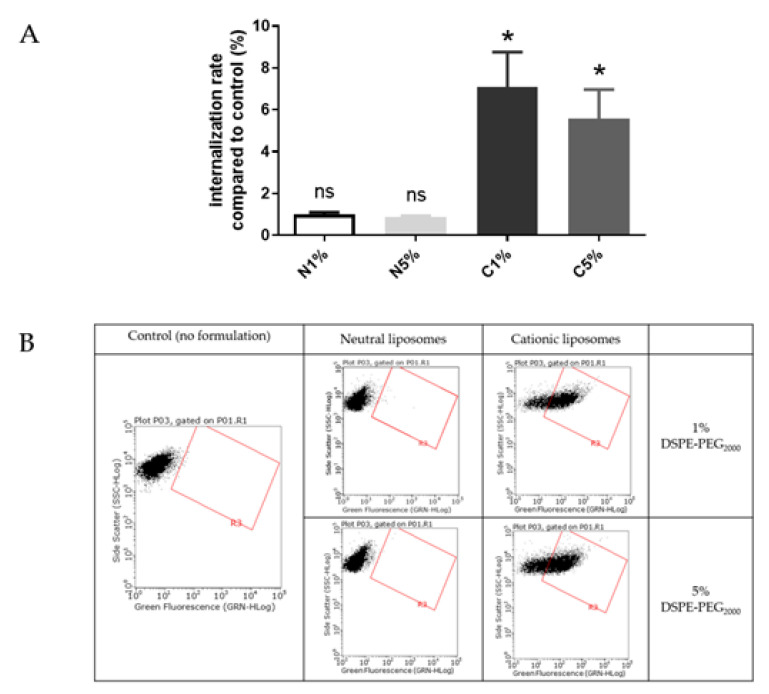
(**A**) Internalization into BeWo cells of neutral liposomes (0.15 mM) and cationic liposomes (0.03 mM) containing 1 % and 5 % PEG. Internalization rates correspond to fluorescence from flow cytometry experiments relative to the control (cells incubated with medium only); data represent the mean  ±  SEM (n  =  3); * indicates a significant difference compared to the control (*p*  <  0.01, Mann-Whitney); (**B**) Flow cytometry dot plots for liposomes internalization evaluation (green fluorescence, DOPE-NBD) after incubation with fluorescent neutral or cationic liposomes (1 or 5 % PEG) at 0.15 mM and 0.03 mM respectively for 4 h. A minimum of 15,000 cells/samples were analyzed.

Liposomes uptake by BeWo cells was also evaluated using FACS (Figure 4). The results are presented as a ratio of the mean fluorescence intensity of each sample compared to the control, i.e., cells incubated with medium only.

Liposomes were incubated with BeWo cells at concentrations determined by MTT tests to maintain 80% viability. After treatment with neutral liposomes at 0.15 mM independent of the PEGylation density, the fluorescence emitted by trophoblasts was equivalent to cells untreated with liposomes. While after treatment with cationic liposomes at 0.03 nM, the trophoblasts significantly gained fluorescence, exhibiting a significant internalization rate. For both neutral and cationic liposomes no influence of the PEGylation density was observed on the internalization of formulation (Figure 4).

Internalization of neutral and cationic liposomes results obtained with the in vitro BeWo cell line model agreed with results from the ex vivo villous placental explants model. Therefore, the BeWo cells can be used as a screening model for further experiment.

**Figure 5:**
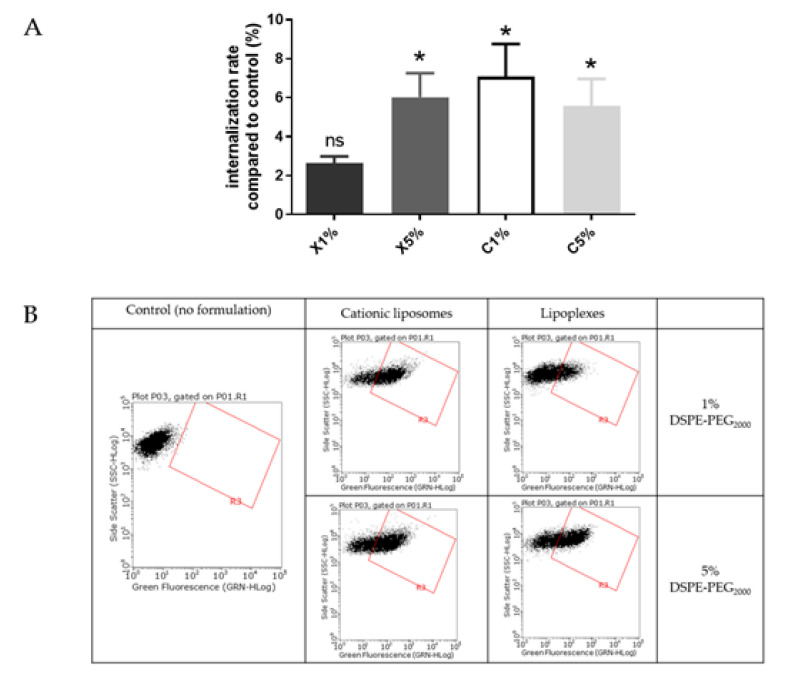
(**A**) Internalization study of cationic liposomes and lipoplexes (0.03 mM) containing 1% and 5% PEG. Internalization rates correspond to fluorescence from flow cytometry experiments relative to the control (cells incubated with medium only); data represent the mean ±  SEM (n  =  3); * indicates a significant difference compared to the control (*p*  <  0.01, Mann-Whitney); (**B**) Flow cytometry dot plots for liposomes internalization evaluation (green fluorescence, DOPE-NBD) after incubation with fluorescent cationic liposomes and lipoplexes (1 or 5 % PEG) at 0.03 mM for 4 h. A minimum of 15,000 cells/samples were analyzed.

These results were also in accordance with previous publications, showing a better internalization of cationic liposomes inside these cells than neutral liposomes [15]. But one publication performed on perfused placenta showed different results: neutral liposomes enhanced transplacental passage of a fluorescent compound, the carboxyfluoresceine, whereas the cationic liposomes prevented its transplacental passage [16]. Neutral liposomes contained similar lipids to the ones used in this study (phosphatidylcholine and cholesterol) but cationic liposomes had a different composition (phosphatidylcholine, cholesterol and stearylamine), and both formulations were unPEGylated. In addition, the fluorescence dye was encapsulated inside the liposomes and not covalently bound to the lipid composing the liposomes, like the DOPE-NBD, which lead to undirect tacking of the formulations. Also, these formulations presented smaller sizes than our liposomes (neutral liposomes = 74.6 +/− 5.4 nm and cationic liposomes = 72.1 +/− 5.0 nm). These divergent results could be explained by the difference in sizes, as it has been shown smaller liposomes were taken up more by the placenta than larger liposomes [16]. Therefore, the lipid composition should be investigated to understand if this parameter influences the internalization in placental cells more than the surface charge or the size. For that purpose, the cationic liposomes internalization was studied in comparison with the lipoplexes internalization in BeWo cells, as they exhibit different surface charge but the same lipid composition (Figure 5). To avoid any toxicity, a concentration of 0.03 mM was chosen to maintain a viability of 80 % for BeWo cells incubated with cationic liposomes or lipoplexes as determined by MTT tests.

Cationic liposomes, whatever the PEGylation percentage, exhibited a significant internalization rate (5–7%) compared to control. Lipoplexes with 5% PEG exhibited a similar rate (6%) of internalization. The lipoplexes with 1% PEG showed a lower cell internalization rate (<3%). During the experiment some aggregation of the lipoplexes with 1% PEG in wells has been observed. We could hypothesize that large aggregates of lipoplexes could not be easily internalized by cells.

As lipoplexes presented an overall neutral surface, a similar absence of internalization rate was expected, as the neutral liposomes. But results showed a significant uptake of lipoplexes with 5% PEG comparable to the cationic liposomes. This could be explained by the similar lipid composition of the cationic liposomes and lipoplexes, containing 50% of the DMAPAP, which has been designed to efficiently transfect nucleic acid inside cells [17]. Therefore, lipid composition appeared to be a better parameter than the surface charge to predict internalization in placental cell, explaining the divergence of results on charge impact from the literature [18,19,20].

To better understand the involvement of the lipid composition in liposomes placental uptake, endocytosis mechanisms of cationic liposomes and lipoplexes were investigated and compared using BeWo cells.

### 2.4. Assessment of Uptake Mechanisms of Liposomes and Lipoplexes by Trophoblasts

To study mechanisms of endocytosis involved in cationic liposomes and lipoplexes placental uptake, four inhibitors were used to inhibit the different pathways of cell endocytosis. Amiloride and derivatives (dimethylamiloride, DMA) modulate the actin cytoskeleton involved in macropinocytosis [21]. Chlorpromazine inhibits clathrin mediated endocytosis [22]. Filipin is specific of caveolae-mediated endocytosis [23]. Dynasore is an inhibitor of the GTPase protein dynamin which is responsible for several endocytic pathways (dynamin dependent endocytosis) [24].

**Figure 6:**
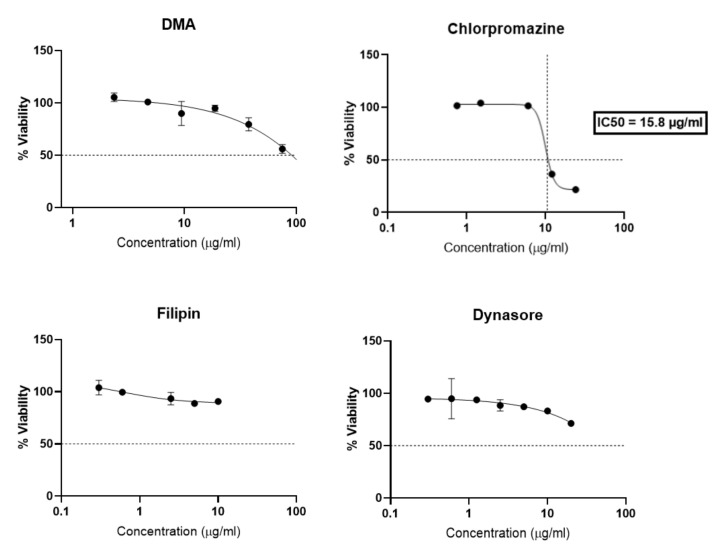
Dose-response curves expressing the viability of BeWo cells after incubation with inhibitors endocytosis (DMA, Chlorpromazine, Filipin, Dynasore) for 4 h. The data represent the results of MTT assay and are expressed as average % cell viability ± SEM against the concentration of the liposomes in mM. Error bars indicate standard deviation for n = 3 experiments.

Compared to previous studies [25,26,27] the different endocytosis inhibitors have been specifically selected for trophoblast, as a matter of non-cytotoxicity, and concentrations and time selected for the assay were chosen based on a viability study (Figure 6). After 4 h of incubation with BeWo cells, chlorpromazine greatly reduced cellular viability with an IC50 of 15.8 µg/mL. DMA, Filipin and Dynasore showed less toxicity since filipin preserved 80% of cell viability, and DMA and Dyansore preserved 50% of cell viability for all concentrations tested. DMA and Dynasore allowed 80% cell viability at 10 µg/mL and 20 µg/mL respectively.

**Figure 7:**
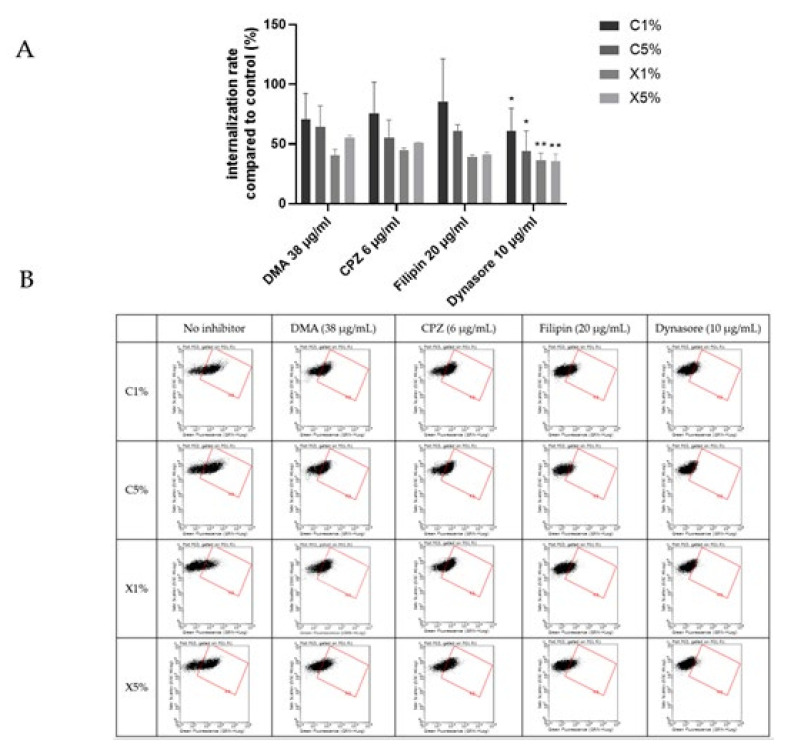
Results of the effect of endocytosis inhibitors on the uptake of cationic liposomes by BeWo cells. Internalization rates correspond to fluorescence from flow cytometry experiments relative to cells incubated with liposomes without inhibitors; data represent the mean  ±  SEM (n  =  3 formulations), * indicates a significant difference compared to the control (*p*  <  0.01, Mann-Whitney) and ** indicates a significant difference compared to the control (*p*  <  0.001, Mann-Whitney); (**B**) Flow cytometry dot plots for liposomes internalization evaluation (green fluorescence, DOPE-NBD) after incubation with fluorescent cationic liposomes and lipoplexes (1 or 5% PEG) at 0.03 mM for 4 h with or without inhibitors dimethylamiloride (DMA), Chlorpromazine (CPZ), Filipin and Dynasore. (**A**) minimum of 15,000 cells/samples were analyzed.

Figure 7 showed an overall decrease of cationic liposomes and lipoplexes uptake by BeWo cells in the presence of the different inhibitors. But only a significant inhibition of the uptake was noticed in presence of the dynasore for all the four formulations. Dynasore inhibits dynamin, which concerns all the pathways of endocytosis except the route of macropinocytosis. Cationic liposomes and lipoplexes are internalized into trophoblasts using several pathways involving dynamic dependent pathways and not micropinocytosis. The implication of different pathways could be expected since liposomes can enter other types of cells through different mechanisms [28,29].

Once again, cationic liposomes and lipoplexes exhibited similar behavior in placental internalization, upholding the influence of lipid composition involvement in the uptake. Breton et al. revealed that DMAPAP lipoplexes enter cells via clathrin-mediated endocytosis and macropinocytosis, but this study is the first to report endocytosis mechanisms of liposomes and lipoplexes containing DMAPAP in placental cells.

Factors influencing the internalization of liposomes have been extensively studied on many cell types [30,31]. But few studies have focused on placental cells especially in combining both placental in vitro and ex vivo models [29,32]. Moreover, studies evaluating endocytosis mechanisms of placental cells concerned other nanoparticle types and not liposomes. One study has evaluated the internalization of gold nanoparticles on placental explants and revealed their translocation through the maternal-fetal barrier by clathrin-mediated endocytosis [33]. Tang et al. have demonstrated that the main placental internalization mechanisms of negatively charged pullulan acetate nanoparticles by the BeWo cell line were pinocytosis and caveolae-mediated endocytosis [34]. More recently, the internalization of positively charged polymeric nanoparticles was related to dynamin dependent processes: mainly clathrin-mediated endocytosis [35]. Therefore, our data provide for the first time a comprehension of liposomes endocytosis mechanisms on placental cells.

## 3. Materials and Methods

### 3.1. Materials

DMAPAP (2-{3-[bis-(3-aminopropyl)-aino]-propylamino}-N-ditetradecylcarbamoyl methyl-acetamide), a cationic lipid was synthesized in our lab [36]. DOPC 18:1 (1,2-dioleoyl-sn-glycero-3-phosphocholine), DSPE-PEG_2000_ 18:0 (1,2-distearoyl-sn-glycero-3-phosphoethanolamine-*N*-[amino(polyethylene glycol)-2000] (ammonium salt)), DOPE-NBD (1,2-dioleoyl sn-glycero-3-phosphoethanolamine-*N*-(7-nitro-2-1,3-benzodiaoxadiazol-4-yl), were purchased from Avanti^®^ Polar Lipids (Avanti, polar lipids, Alabaster, AL, USA). Analytical grade ethanol isopropanol and methanol were purchased from Carlo Erba Reagents^®^ (Carlo Erba reagents, Val de Reuil, France). Dimethyamiloride (DMA), ConcanavalinA, Filipin III, Dynasore, EDTA (Ethylenediaminetetraacetic acid), cholesterol, NaCl, penicillin streptomycin, sodium alginate (AA), sterile DPBS (10×) (Dulbecco’s phosphate buffer solution), DMEM (1×) (Dulbecco’s modified eagle’s medium with F-12 Nutrient Mixture), DMEM/F-12 (1×), 0.05% trypsin-EDTA (1×), fetal bovine serum and MTT (3-(4,5-dimethylthiazol-2-yl)-2,5-diphenyl tetrazolium bromide) were purchased from Merck ^®^ (Merck, Saint Quentin Fallavier, France). Paraformaldehyde (PFA) was purchased from Electron Microscopy Sciences (Electron microscopy science, Hatfield, United Kingdom). Monoclonal mouse anti-human primary antibody against Cytokeratin 7 (CK7) (Clone OV-TL 12/30) was purchased from Dako^®^ (Dako, Glostrup, Denmark), and goat anti-mouse secondary antibody Alexa Fluor^®^ 555 against mouse IgG, as well as DAPI were purchased from Invitrogen^®^. Bovine serum albumin (BSA) was purchased from Interchim (Montluçon, France). A Millipore^®^ Milli-Q system (Millipore, Fontenay-sous-Bois, France) was used to obtain purified water. Fluorescent mounting medium was purchased from Agilent (Agilent, Santa-Clara, CA, USA).

Mini extruder was purchased from Avanti Polar Lipids (Avanti polar lipids, Alabaster, USA), Centrifuge model Rotanta 460 RF was purchased from Hettich (Hettich, Tullingen, Germany). Vibratome and confocal laser microscope are provided by Leica (Leica, Wetzlar, Germany). The Nano Zeta Sizer (ZS) was purchased from Malvern instruments (Malvern instruments, Worcestershire, UK). HPLC apparatus was bought from Shimadzu (Shimadzu, Kyoto, Japan). The HPLC column Kromasil (C-18) was purchased from AkzoNobel (AkzoNobel, Amsterdam, Netherland).

### 3.2. Liposomes and Lipoplexes Preparation

Liposomes were prepared as previously described [8,9]. Briefly, the lipids with different molar ratios (DMAPAP/DOPE/DSPE-PEG2000/DOPE-NBD, 50/44-48/5-1/1) for cationic liposomes (C) and (DOPC/Chol/DSPE-PEG2000/DOPE-NBD, 60/34-38/5-1/1) for neutral liposomes (N) were dissolved in chloroform. Then chloroform was evaporated by rotary evaporation, and the formed thin film was hydrated using 150 mM NaCl solution to a final lipid concentration of 5 mM.

The resulting suspension was extruded (11 passages) using the Avanti^®^ Polar Lipids Mini-Extruder set through Whatman^®^ Nuclepore™ Track-Etched polycarbonate membranes (ø = 19 mm) of 0.8, 0.4, 0.2, and 0.1 μm pore size to obtain liposomes of a smaller and more homogenous size distribution. Lipoplexes were prepared by mixing equal volumes of the cationic liposomal suspension with a non-coding siRNA and sodium alginate (AA) in 150 mM NaCl, and then rapidly vortexed. The N/P charge ratio was 4, calculated using the molar ratio of charges of DMAPAP, siRNA and AA. The calculation used the molar ratio of charges of DMAPAP, siRNA and AA; DMAPAP has three positive charges per molecule while siRNA has 3.08 nmol negative charges per microgram and AA has 5.05 nmol negative charges per microgram. Each formulation was manufactured with and without 1 % of fluorescent lipid DOPE-NBD, to investigate if the fluorescence had any influence on the results obtained in the study and details of formulations are presented in Table 3.

Nano ZS (Malvern Instruments Ltd., Malvern, UK) was used to determine the mean particle hydrodynamic diameter (Z-average), polydispersity index (PdI) and Zeta potential. A 1/100 dilution of liposomes or lipoplexes suspension was carried out in 20 mM NaCl prior to size measurement and to zeta potential assessment. A RiboGreen^®^ assay was used to determine the encapsulation efficiency of siRNA according to the manufacturer’s instructions (Invitrogen, Carlsbad, CA, USA).

### 3.3. In Vitro Evaluation of Liposomes and Lipoplexes Uptake by Trophoblasts

#### 3.3.1. Cell Culture

BeWo cells, a human choriocarcinoma derived cell line, were used as an in vitro placental model for the study of liposomal cytotoxicity and uptake. Cells were cultured in T75 flasks using phenol red free DMEM/-F12 (1:1, *v*/*v*) medium completed with 10% fetal bovine serum (FBS) and 1% penicillin-streptomycin. Cells were incubated at 37°C and 5% CO_2_, renewing the medium every 24–48 h.

#### 3.3.2. MTT Cytotoxicity Assay

MTT assay was performed to test cell viability after incubation with the different liposomal formulations. 96-well plates were seeded with 7,500 cells per well containing 100 μL of complete medium. Plates were incubated for 24 h at 37 °C and 5% CO_2_ to allow cell adhesion. Medium was then replaced with 100 μL of fresh medium containing liposomes. After 24 h of incubation, the medium in each well was discarded and replaced by 100 μL of a 0.5 mg/mL MTT solution in complete medium. After additional 4 h of incubation, the MTT solution was removed and 100 μL of DMSO were added to each well to dissolve formazan crystals. After 10 min agitation to allow complete dissolution, absorbance at λ = 560 nm was measured with a multimode plate reader TECAN Infinite^®^ F200 Pro.

#### 3.3.3. Formulations’ Uptake Evaluation Using Fluorescence Activated Cell Sorting (FACS)

Internalization study was performed using Fluorescence activated cell sorting analysis (FACS) on BeWo cells also. 12-well plates were seeded with 200,000 cells per well containing 1 mL of complete medium. Plates were incubated for 24 h at 37 °C and 5% CO_2_ to allow adhesion. After 24 h, medium was replaced by 1 mL of fresh medium containing liposomes at a concentration of 0.15 mM total lipid. Cells were then incubated for 24 h. After incubation cells were rinsed with 0.5 mL of sterile DPBS and detached with 0.5 mL of 0.05% trypsin-EDTA. Following wells scraping, cell suspensions were retrieved and centrifuged at 200 rpm for 5 min at 4 °C. Supernatant was discarded and cell pellet was suspended in 400 μL of PBS 1× containing 0.5% BSA and 2 mM EDTA. Samples were kept in ice until flow cytometry measurements were acquired.

### 3.4. Ex Vivo Evaluation of Liposomes and Lipoplexes Uptake by Villous Placental Explants

#### 3.4.1. Ethical Statement

Term placenta between from 37 to 41 weeks of pregnancy were obtained after C-section from the patients of Cochin Port-Royal maternity units, after receiving their written informed consent. The protocol was approved by the institutional review board “Comité de Protection des Personnes 2015-mai-1390”.

#### 3.4.2. Incubation of Liposomes and Lipoplexes with Villous Placental Explants

Collected placenta are then dissected to isolate villi of 1 cm^3^ approximately. Villi are then kept in culture for 24 h in DMEM completed with 10 % FBS, 1% glutamine and 1% Penicillin-Streptomycin mixture.

After dissection of the placenta to isolate villous placental explants, villi are suspended on needles inside the wells and liposomes are then incubated at various concentrations (0.15, 0.3 and 0.5 mM) and kept for 24 h in culture at 37 °C and 5% CO_2_. After villi are washed with 1% sterile DPBS 1× fixed with 4% PFA, then diluted to 1% PFA or quickly snap frozen in liquid nitrogen to be, respectively, used for confocal microscopy imaging or HPLC analysis.

#### 3.4.3. Qualitative Analysis of Liposomes Uptake by Confocal Microscopy

After fixation in 1% PFA, villi are washed with PBS 1× and are embedded in agarose for immunostaining. Sample slices of 100 μm thickness are cut using a Vibratome (Technical Products International, Maryland Heights, MO, USA). Slices are permeabilized and saturated with a 0.1% Triton X-100, 7% bovine serum albumin (BSA) solution for 3 h at room temperature under gentle agitation. Immunolabelling of the tissues was realized overnight using mouse antibodies against the human cytokeratin 7 (CK7). Immunostaining with secondary antibody was carried out for 2 h using a goat anti-mouse antibody coupled to Alexa Fluor^®^ 555. Nuclei were stained with DAPI at 1/15,000 dilution for 10 min under agitation. Slices were observed with a TCS SP2 confocal microscope (Leica, Wetzlar, Germany) localized at the platform of microscopy of the school of pharmacy, INSERM UMS 025—CNRS UMS 3612, Université Paris Cité.

#### 3.4.4. Quantitative Analysis of Liposomes Uptake by HPLC Method

After snap freezing in liquid nitrogen, villi can be stored at −80 °C. For analysis with HPLC, villi are thawed at room temperature. A liquid-liquid extraction is performed, samples containing DOPE-NBD are analyzed by HPLC according to the protocol previously described [14].

To extract the fluorescent probe, DOPE-NBD from placental tissue the liquid-liquid extraction was performed as follows. Each explant was sliced with a scalpel. Then 400 µL of MilliQ water and 100 µL of 10 % Triton X-100 was added to the mixture and vortexed for 30 s. Each sample was sonicated for 10 min for two cycles and vortexed in-between. The mixture was transferred to a polypropylene tube and 3 mL of chloroform were added. Each sample was vortexed for 20 min, followed by 10 min of centrifugation at 2000 rpm for 10 min. The organic phase was collected in a round bottom flask and the process was repeated with 3 mL of chloroform. The organic solvent was evaporated at low pressure on a rotary evaporator at room temperature. The collected lipid film in the flask was reconstituted in 500 µL of ethanol and filtered on a 0.2 µm cellulose acetate Nalgene^®^ filter and 50 µL was injected in the HPLC.

The HPLC was carried out on a Shimadzu LC-20AD high perform liquid-chromatography completed with a quaternary pump, mobile phase degaser and a C18 Kromasil column (5 µm, 4.6 mm × 250 mm, 100 Å) and protected with a pre-column (20 mm × 3.9 mm) filled with the same stationary phase and operated at 40 °C. The detection was performed using a Shimadzu fluorescence detector (RF-10AXL) operating at 465 nm and 535 nm for excitation and emission wavelengths, respectively. The HPLC monitoring and data acquisition was performed by using LabSolutions software. The gradient mobile phase was composed of water, methanol and isopropanol. The first 5 min of initial conditions of mobile phase were 10% water, 80% methanol and 10% isopropanol. Then it was changed in 5 min to 40% methanol and 60% isopropanol and maintained for 5 more minutes. Finally, the mobile phase returned to the initial conditions of 10% water, 80% methanol and 10% isopropanol in 5 min and were maintained for 5 more minutes. The total time of the elution gradient was 25 min with a flow rate of 1 mL/min.

## 4. Conclusions

This study aims to better understand the characteristics of liposomes and lipoplexes, which influence their penetration inside placental cells, such as the amount of PEG at the surface of the formulations, their surface charge, and their lipid composition The general goal of this study is to understand the effect of the nanocarrier design on its safety and efficacy, and to select the appropriate one for each application: to treat the mother or to treat the placenta and preventing transplacental passage of the encapsulated API.

Our results highlighted the major influence of the lipid composition compared to the surface charge and the PEGylation density on the cellular internalization. Cationic liposomes and lipoplexes containing a non-coding siRNA, showed similar behavior with internalization driven by dynamin dependent pathways, which confirmed the influence of the lipid composition on the placental uptake of the liposomes.

The methodology used in this work has proven to be useful to investigate nanoparticles interaction with the placental barrier using in vitro and ex vivo complementary models with Human origin. This methodology could be applied to further studies to assess lipids nanoparticles with different compositions. It will also benefit to standardize protocols for the development of new formulations with more complex surface characteristics to deliver therapeutics to the placenta. In this work blank nanomedicines were used to investigate their biological behavior towards the placenta. This study gives preliminary information which could be useful for further therapeutics development by encapsulating APIs within the liposomes. Hopefully, this study will lead to a better understanding of the use of nanoparticles during pregnancy and can pave the way for safer treatment of pregnant woman.

## Figures and Tables

**Table 1:** Liposomes’ and lipoplexes’ physicochemical characteristics expressed as mean ± SD of three batches for each formulation.

Formulation	Composition	% of DSPE-PEG2000	Z-Average (nm ± SD)	PdI	Zeta Potential (mV ± SD)
Cationic liposomes	DMAPAP/DOPE/DSPE-PEG2000/DOPE-NBD	5	108.5 ± 0.9	0.07	31 ± 0.7
		1	106.3 ± 2.2	0.08	36.1 ± 1.1
Neutral liposomes	DOPC/Chol/DSPE-PEG2000/DOPE-NBD	5	130.3 ± 3.7	0.07	3.2 ± 7.2
		1	124.9 ± 1.1	0.11	−10.3 ± 0.3
Lipoplexes	DMAPAP/DOPE/DSPE-PEG2000/DOPE-NBD + non-coding siRNA + sodium alginate	5	199.0 ± 13.3	0.19	10.7 ± 5.5
		1	189.6 ± 17.4	0.23	6.7 ± 1.5

**Table 2:** DOPE-NBD quantification in placental tissue after incubation with neutral and cationic liposomes at various concentrations. The amount of DOPE-NBD is normalized to their weight.

Liposomes Incubated	Sample Name	Weight of Villi (mg)	Total Lipid Concentration (µmol/L)	DOPE-NBD Concentration (µg/L)	DOPE-NBD Concentration Extracted (µg/L)	Mass of DOPE-NBD Extract (µg)	Amount of DOPE-NBD in 1 g of Villi (µg)
**N5%**	C3	18	150	136	<1	nd	nd
	C7	25	300	272	<1	nd	nd
	C11	12	500	454	2.0	0.001	0.040
**C5%**	B3	12	150	136	28.1	0.014	1.172
	B7	9	300	272	69.8	0.035	3.875
	B11	17	500	454	145.7	0.073	4.285

**Table 3:** Composition of liposomes and lipoplexes included in the study.

Type of Liposomes	Composition	% of DSPE-PEG2000	% of DOPE-NBD
Cationic liposomes	DMAPAP/DOPE/DSPE-PEG2000/DOPE-NBD	5	1
		5	0
		1	1
		1	0
Neutral liposomes	DOPC/Chol/DSPE-PEG2000/DOPE-NBD	5	1
		5	0
		1	1
		1	0
Lipoplexes	DMAPAP/DOPE/DSPE-PEG2000/DOPE-NBD + non-coding siRNA + sodium alginate	5	1
		5	0
		1	1
		1	0

## Data Availability

Not applicable.
